# Effect of TiO_2_ nanoparticles incorporation on antibacterial properties and shear bond strength of dental composite used in Orthodontics

**DOI:** 10.1590/2177-6709.22.5.067-074.oar

**Published:** 2017

**Authors:** Ahmad Sodagar, Mohamad Sadegh Ahmad Akhoundi, Abbas Bahador, Yasamin Farajzadeh Jalali, Zahra Behzadi, Farideh Elhaminejad, Amir Hossein Mirhashemi

**Affiliations:** 1 Tehran University of Medical Sciences, Dental Research Center, Dentistry Research Institute, Faculty of Dentistry, Department of Orthodontics (Tehran, Iran).; 2 Tehran University of Medical Sciences, Faculty of Medicine, Department of Microbiology (Tehran, Iran).; 3 Ilam University of Medical Sciences, Faculty of Dentistry, Department of Orthodontics (Ilam, Iran).; 4 Tehran University of Medical Sciences, Faculty of Dentistry (Tehran, Iran).

**Keywords:** Titanium dioxide, nanoparticles, Dental bonding, Antimicrobial orthodontic adhesive, Shear strength

## Abstract

**Introduction:**

Plaque accumulation and bond failure are drawbacks of orthodontic treatment, which requires composite for bonding of brackets. As the antimicrobial properties of TiO_2_ nanoparticles (NPs) have been proven, the aim of this study was to evaluate the antimicrobial and mechanical properties of composite resins modified by the addition of TiO_2_ NPs.

**Methods:**

Orthodontics composite containing 0%, 1%, 5% and 10% NPs were prepared. 180 composite disks were prepared for elution test, disk agar diffusion test and biofilm inhibition test to collect the counts of microorganisms on three days, measure the inhibition diameter and quantify the viable counts of colonies consequently. For shear bond strength (SBS) test, 48 intact bovine incisors were divided into four groups. Composites containing 0%, 1%, 5% and 10% NPs were used for bonding of bracket. The bracket/tooth SBS was measured by using an universal testing machine.

**Results:**

All concentration of TiO_2_ NPs had a significant effect on creation and extension of inhibition zone. For *S. mutans* and *S. sanguinis,* all concentration of TiO_2_ NPs caused reduction of the colony counts. Composite containing 10% TiO_2_ NPs had significant effect on reduction of colony counts for *S. mutans* and *S. sanguinis* in all three days. The highest mean shear bond strength belonged to the control group, while the lowest value was seen in 10% NPs composite.

**Conclusions:**

Incorporating TiO_2_ nanoparticles into composite resins confer antibacterial properties to adhesives, while the mean shear bond of composite containing 1% and 5% NPs still in an acceptable range.

## RESUMO

**Introdução:**

o acúmulo de placa e as descolagens de braquetes são algumas desvantagens presentes no tratamento ortodôntico, no qual se requer o uso de materiais compósitos para a colagem dos braquetes.

**Objetivo:**

tendo em vista que as propriedades antimicrobianas das nanopartículas (NPs) de TiO_2_ já foram confirmadas, o objetivo do presente estudo foi avaliar as propriedades antimicrobianas e mecânicas de resinas compostas modificadas pela adição de NPs de TiO_2_.

**Métodos:**

compósitos ortodônticos contendo 0%, 1%, 5% e 10% de NPs foram preparados. Cento e oitenta discos de compósito foram preparados para o teste de eluição, o ensaio de difusão em ágar por disco, e o ensaio de inibição da formação de biofilme, para se calcular as contagens de microrganismos ao longo de três dias, medir o diâmetro da inibição e, consequentemente, quantificar as contagens de colônias viáveis. Para o teste de resistência da colagem ao cisalhamento (SBS), 48 incisivos bovinos intactos foram divididos em quatro grupos, nos quais os compósitos contendo 0%, 1%, 5% e 10% de NPs foram utilizados para colagem dos braquetes. A SBS da interface braquete/dente foi medida em uma máquina universal de ensaios.

**Resultados:**

todas as concentrações de NPs de TiO_2_ apresentaram efeito significativo na formação e na extensão da zona de inibição. Para o *S. mutans* e o *S. sanguinis*, todas as concentrações de NPs de TiO_2_ causaram redução na contagem das colônias. O compósito contendo 10% de NPs de TiO_2_ apresentou uma diminuição significativa na contagem de colônias de *S. mutans* e *S. sanguinis* durante os três dias. A média mais alta da SBS foi observada no grupo controle, enquanto o valor mais baixo foi observado para o compósito com 10% de NPs.

**Conclusões:**

a incorporação de nanopartículas de TiO_2_ nas resinas compostas lhes conferiu propriedades antibacterianas, e o valor médio da SBS das resinas contendo 1% e 5% de NPs apresentou-se dentro de uma faixa aceitável.

## INTRODUCTION

Bonding technique with resin-based composite as an adhesive agent has been primarily used in Orthodontics for securing orthodontic brackets to the surface of the teeth. Unfortunately, in spite of the fact that bonding technique have many advantages - such as high esthetic and simple procedure -, still have some drawbacks such as plaque accumulation, development of white spot lesions and bond failure, which cause prolonging the treatment course, imposing high cost, consuming more chair time and a less than optimal esthetic result occurs after treatment, due to demineralization of enamel adjacent to the brackets, specially around the bracket margin, due to the surface exposed to composite.^1^ ^-^ ^6^

Several methods have been used to inhibit biofilm growth, which contributes to dental caries. For instance, one group of such efforts has been evaluation of effectiveness of incorporating different antimicrobial agents in the adhesives; two of the most common examples are fluoride and chlorhexidine.^7^ ^-^ ^12^

Today one of the most important advances in dental material field is the application of nanotechnology to resin composites. Many studies investigated the effect of antimicrobial nanoparticles incorporated into composite resins to prevent plaque accumulation and bacterial adhesion. Nanoparticles are believed to penetrate into the cell wall of bacteria efficiently due to their smaller size, effectively exerting their antibacterial properties.^13^ ^,^ ^14^ A study evaluated the antibacterial effect of composite with different concentration of incorporated nanosilver, which has been used in Medicine as an antimicrobial agent. The result of this study showed that nanosilver containing composite could confer surface antibacterial activity without significant difference on shear bond strength.^15^ Another study demonstrated that addition of nanosilver significantly decreased the shear bond strength of orthodontic brackets; however, the obtained mean value was still higher than the estimated force required for orthodontic treatment.^16^ Silver/hydroxyapatite nanoparticles were shown to decrease the shear bond strength as their concentration increased.^17^ Evidence shows that addition of ZnO nanoparticles to composite resin increases the mechanical properties such as compressive strength and flexural modulus.^18^ ZnO nanoparticles also possess antimicrobial properties and inhibit the formation of biofilm.^19^ Chitosan is also used in many industries and is available in nano and microforms.^20^ ^,^ ^21^ Recently, it has been shown that chitosan has antibacterial activity against a wide range of bacteria. Chitosan nanoparticles also enhance the antibacterial properties of composite resins without compromising their shear bond strength.^22^ It has also been demonstrated that chitosan nanoparticles in conjunction with ZnO nanoparticles could improve the antimicrobial effect of composite resins, while the obtained shear bond strength values were approximately similar to conventional orthodontic composites.^23^

Antimicrobial properties of TiO_2_ nanoparticles and their application in Medicine, Dentistry and other science have been widely known. Haghi et al^24^ evaluated the antimicrobial effect of TiO_2_ NPs on pathogenic strain of *E. coli* and they showed that the TiO_2_ NPs cause little pores in bacterial cell walls, leading to increased permeability and cell death. Based on this study, nano-TiO_2_ has efficient antibacterial effect and can be used as an antibacterial agent for different purposes. 

We decided to assess the effect of incorporating different percentages (wt %) of TiO_2_ nanoparticles into composite resin to obtain a composite with excellent antimicrobial properties and sufficient bond strength to enamel surface for use in Orthodontics.

## MATERIAL AND METHODS

### Nanocomposite preparation

For the fabrication of composite containing different concentration of TiO_2_ NPs, 400 mg of TiO_2_ NP powder was manually mixed with 3600 mg of Transbond XT composite (3M Unitek, Monrovia, CA, USA), in order to obtain 4000 mg of composite containing TiO_2_NPs with 10% concentration. To fabricate composite containing 5% TiO_2_ NPs, 1200 mg of composite containing 10% TiO_2_ NPs was mixed with 1200 mg of plain composite. To fabricate composite containing 1% TiO_2_NPs, 240 mg of 10% TiO_2_ NPs was mixed with 2160 mg of plain composite. Thus, 2560 mg of composite containing 10% TiO_2_NPs, 2400 mg of composite containing 5% TiO_2_ NPs and 2400 mg of composite containing 1% TiO_2_ NPs were thus obtained.

### Shear bond strength test

Forty-eight bovine central incisors with no visible cracks or caries were disinfected in 0.5% chloramine-T solution (4°C) for one week. Specimens were randomly divided into four groups (n = 12) of composites with 0%, 1%, 5% and 10% NP content. Buccal surfaces were cleaned with a prophylaxis brush without powder, rinsed and dried; 35% phosphoric acid (Ultra-Etch, Ultradent, USA) was then applied to the buccal surfaces for 30 seconds, followed by 30 seconds of rinsing and gentle air drying. A uniform, thin layer of adhesive (3M Unitek, USA) was applied to the buccal surfaces and light cured (Woodpecker, UK) for 10 seconds after placing the stainless steel orthodontic brackets (Standard edgewise, 0.018-in slot, 12.62 mm^2^ base area).

All specimens were thermocycled (Vafaei Industrial, Iran) for 1000 rounds in 24 hours to simulate oral environment. Each cycle consisted of 15 seconds of immersion in 5°C water bath, 10 seconds of dwell time and 15 seconds of immersion in 55°C water bath. The thermocycled teeth were then fixed to the corners of 2.5-cm diameter metal molds using rectangular wire and then the molds were filled with self-curing acrylic resin (Acropars, Iran) up to the level of the cementoenamel junction. 

**Figure 1:**
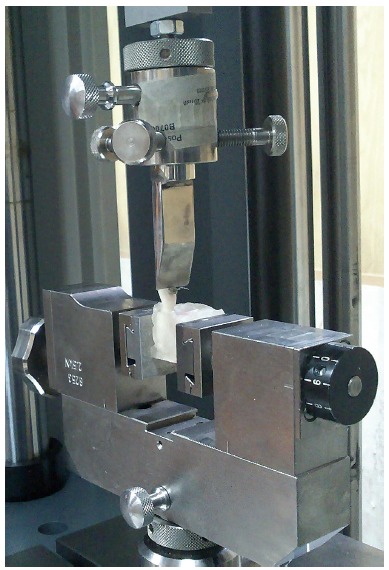
A 0.6-mm metal blade was used to apply shear force to the composite interface.

The shear bond strength (SBS) was measured using Roell-7060 universal testing machine (Zwick/Roell, Germany). Specimens were positioned in such way that the bracket base was parallel to the direction of the applied force. A 0.6-mm metal blade was used in an inciso-gingival direction at a crosshead speed of 0.5 mm/min to apply shear force to the composite interface (Fig 1). The obtained value (N) was divided by the bracket surface area (mm^2^) to calculate the SBS in megapascals. Each tooth and bracket complex was then checked under a stereomicroscope (Nikon, SMZ800, Japan) at 10 X magnification, to score the amount of remaining adhesive, by using the adhesive remnant index (ARI) as follows: 0 = no adhesive on bracket, 1 = < 25% adhesive on bracket, 2 = 25-50% adhesive on bracket, 3 = 50-75% adhesive on bracket and 4 = 75-100% adhesive on bracket.

### Antimicrobial test

Preparation of bacterial suspensions: *Streptococcus mutans* ATCC25175, *Streptococcus sanguinis* ATCC10556 and *Lactobacillus acidophilus* ATCC4356 were supplied in liophilised form and incubated in broth, in anaerobic and 37°C conditions for 48 hours. 10^8^ CFU/ml microorganism suspensions were prepared by spectrophotometer for determining the antimicrobial effect of TiO_2_ NPs. 

Optical density for *L. acidophilus* in 600 nm is 1 (OD = 1); which is 10^8^ cells per ml. This density was then diluted ten times and inoculated on BHI (brain-heart infusion) agar. Optical density for *S. mutans and S. sanguinis* was OD = 0.2.

### Composite disc preparation

**Figure 2:**
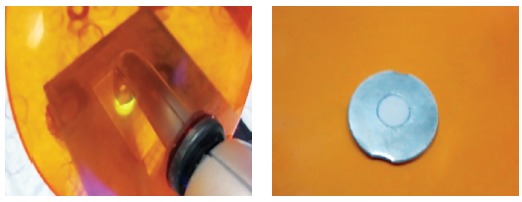
Composite disc preparation.

Metal washers were placed on a glass slab and inside the washers, composite was applied with 5-mm diameter and 0.64-mm thickness. Next, a thin layer of bonding agent was applied on the samples. The reason behind selection of this particular diameter of metal washers was their equal size to bracket base. A smooth thin glass slob was placed on top of the samples and mildly compressed, to obtain a smooth composite surface with no porosity, and also to obtain equal thickness in all samples. Each sample was light-cured for 30 seconds. Then, the samples were separated from the washers and sterilized in Iran’s Nuclear Science and Technology gamma radiation center with 25 KGy dosages (Fig 2).

### Biofilm inhibition

Three-day biofilms were generated on composite discs (n = 36) using 24-well plates. Each well was inoculated with adjusted bacterial inoculum. Biofilms were grown at 37°C. At the end of the third day, each disc was rinsed with sterile saline solution to remove loosely adsorbed proteins and biofilm matrix residues. To count the colony forming units (CFUs) responsible for biofilm formation, specimens were sonicated in sterile saline solution and then vortexed. CFU/ml of the microorganism present in the suspension was counted with drop-plate method using rapid dilution in microtiter plates.

### Disc Agar Diffusion test (DAD)

Antibacterial activity of discs via solubility and diffusion of TiO_2_ NPs was examined by DAD. Composite discs (n = 36) were placed, 2 cm apart, on BHI agar plates, which were inoculated with a 200 µL bacterial solution (≈10^8^CFU/mL) by a sterile swab. After 48-hour incubation, the bacterial growth inhibition diameter was optically measured.

### Antibacterial properties of eluted components

**Figure 3:**
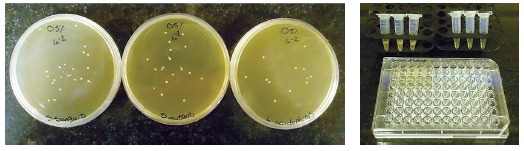
Antibacterial properties of eluted components test.

This test was used to assess the antimicrobial activity of TiO_2_ NPs released from composite discs. Composite discs were placed in tubes containing 0.5 ml of BHI broth. After 3, 15 and 30 days, the discs were transferred to 15-ml plastic tubes and 50 µL of the bacterial culture was added to each tube containing 5 ml of TSA (final concentration of 10^5^ CFUs/ml in 1 ml of medium). The tubes were shaken at 300 rpm at 37^o^C for 24 hours. The obtained suspension was serially diluted in microtiter plates and spread cultured in TSA. The bacterial colonies (CFUs/ml) were counted using drop-plate method (Fig 3). 

**Figure 4:**
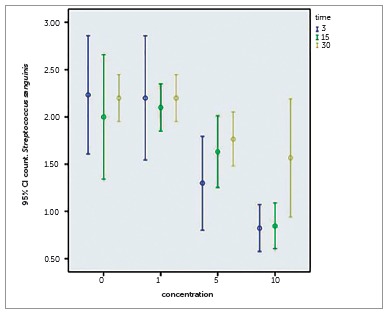
Colony counts of *Streptococcus sanguinis* on the following composites: 0%, 1%, 5% and 10% NPs containing composite discs in Eluted Component Test.

**Figure 5:**
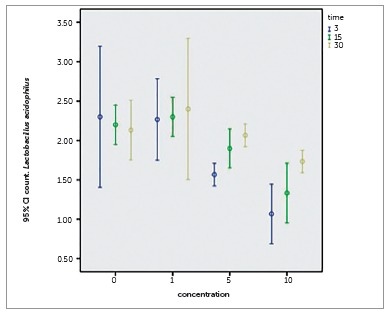
Colony counts of *Lactobacillus acidophilus* on the following composites: 0%, 1%, 5% and 10% NPs containing composite discs in Eluted Component Test.

### Statistical analysis

Shear bond strength test results were analyzed using one-way ANOVA followed by *post-hoc* Tukey’s HSD test. The Kruskal-Wallis test was also applied to analyze the ARI results. Antimicrobial test results were analyzed with multiple statistical tests. One-way ANOVA was first used for biofilm inhibition test, followed by Tukey HSD test. Kruskal-Wallis test was used to analyze data attained from DAD test. Two-way ANOVA was first used to analyze day × concentration relation in eluted components test. For those groups with significant difference, one-way ANOVA was used.

## RESULTS

### Biofilm inhibition test

Mature biofilm on four different composite groups were recorded after 3 days. Descriptive results are shown in Table 1. All tests were carried out three times for each group. Kruskal-Wallis test showed that *S. mutans* and *S. sanguinis* colonies were meaningfully lowered in all three groups of composites containing NPs (*p*< 0.05). However, *L. acidophilus* colonies were meaningfully lowered only in 10% NP containing composite (*p*< 0.002). 

**Table 1:** Colony counts (CFU/mm^2^) in 1%, 5% and 10% NPs containing composite and control group, in biofilm inhibition test.

Percent	Microorganisms	n	Minimum	Maximum	Mean	S.D.
0	S. mutans biofilm	3	761	981	889.67	115.14
	*S. sanguinis* biofilm	3	523	701	630.6667	94.69072
	*L. acidophilus* biofilm	3	72	110	89	19.313
1	S. mutans biofilm	3	391	472	435.33	41.041
	*S. sanguinis* biofilm	3	168	201	181.6667	17.21434
	*L. acidophilus* biofilm	3	39	66	54	13.748
5	S. mutans biofilm	3	112	158	133	23.259
	*S. sanguinis* biofilm	3	41	52	47	5.56776
	*L. acidophilus* biofilm	3	27	29	28	1
10	S. mutans biofilm	3	11	18	14	3.606
	*S. sanguinis* biofilm	3	1.3	2.2	1.7333	4.5092
	*L. acidophilus* biofilm	3	5	9	7	2

### DAD test

In all three repeated tests, all three groups of composites containing NPs had a significant diameter of bacterial growth inhibition for all three microorganisms. Kruskal-Wallis test did not show any significant difference between 1%, 5% and 10% NPs groups. The results are illustrated in Table 2.

**Table 2:** Microorganism inhibition diameter (millimeter) by NPs diffusion.

Microorganisms	Percent	n	Minimum	Maximum	Mean	S.D.
*S. mutans*	0	3	0	0	0	0
	1	3	6	7	6.67	0.577
	5	3	7	8	7.67	0.577
	10	3	10	13	11.33	1.528
*S. sanguinis*	0	3	0	0	0	0
	1	3	6	8	7.33	1.155
	5	3	6	9	8	1.732
	10	3	12	13	12.67	0.577
*L. acidophilus*	0	3	0	0	0	0
	1	3	6	9	7.67	1.528
	5	3	6	9	7.67	1.528
	10	3	9	11	10.33	1.155

**Table 3:** Descriptive data of shear bond strength (MPa) in groups.

Group	n	Minimum	Maximum	Mean	S.D.
Control	12	22.15	44.52	34.47	6.73754
1% NP	12	10.33	24.65	18.17	4.6564
5% NP	12	3.57	23.14	13.9	6.00256
10% NP	12	0	8.28	3.51	3.28044

### Eluted components test

For *S. mutans* no significant difference was found for any of the groups in any day (*p*= 0.144). Groups of composites containing more than 5% NPs on all days significantly reduced colony counts of *S. mutans* (*p*< 0.001). Statistically differences in all three days were found for *S. sanguinis* (*p*= 0.004) and *L. acidophilus* (*p*= 0.020) so each of the categories were analyzed by One-way ANOVA, which showed no significant difference for *L. acidophilus* on different days, and was only statistically significant in groups of composites containing more than 5% NPs for *S. sanguinis* on day 30 (*p*< 0.005).

### Shear bond strength

Descriptive results showed that the highest mean SBS belonged to the control group, while the lowest value was seen in 10% NP composite group. 

One-way ANOVA revealed a statistically significant difference in SBS among the groups (*p*= 0.000). 

Pairwise comparison of groups revealed that the tooth/bracket SBS was significantly higher in the control group and the 1% NP group than the group containing 10% NP (*p*< 0.005).

The Kruskal-Wallis test did not show any significant difference among groups in terms of the ARI (*p*= 0.823). 

## DISCUSSION

The application of nanotechnology in dental composite resins has been introduced to enhance the long-term antimicrobial properties and provide superior mechanical strength simultaneously.^13^ ^,^ ^25^

This research assessed the antibacterial effect and shear bond strength of composite resins containing different percentage of TiO_2_ nanoparticles. The result demonstrated that bacterial biofilm inhibition in composite containing NPs is significantly greater than conventional composite resins. This effect increased as the percentage of NPs in the composites increased in such a way that the composite containing more than 5% NPs significantly reduced *S. mutans* and *S. sanguinis*. However this had no effect on biofilm inhibition of *L. acidophilus,* which help the development of caries and are found in more advanced lesions. Therefore, the biofilm containing these microorganisms is very resistant and is not affected by adding NPs to the composite resins. This finding is in agreement with Poosti et al,^26^ which investigated the inhibition of bacterial biofilm only on *S. mutans* and showed that the inhibition is more prominent with composite containing 1% TiO_2_ (standard P_25_) NPs.

For other nanoparticles such as Ag, higher antibacterial effect than conventional composite has been shown only in direct contact with *S. mutans*. However, bacterial growth was not significantly different in composites with or without NPs when BHI containing *S. mutans* was used^15^. On the other hand, some other studies proved that nanosilver and nanosilica filler-containing composites prevent enamel demineralization around orthodontic bracket, and showed silver NPs have antimicrobial effect against refractory bacteria.^27^ ^,^ ^28^ ^,^ ^29^ Silver NPs create a dark grey color change in composites, which defies the esthetic purposes.

Other nanoparticles - such as chitosan, ZnO and combination of chitosan and ZnO - in dental composites cause inhibition of bacterial biofilm, and this effect is increased as the percentage of NPs increases in composite. Although the greatest effect was seen in composite containing 10% NPs on the first day of testing,^19^ ^,^ ^23^ the shear bond strength of composite was reduced as the percentage of NPs increased. The obtained shear bond strength in <5% NPs groups were in an acceptable range.^22^

The results of eluted component test showing continuity of antibacterial effect indicated significant reduction of the colony count of *S. mutans* and *S. sanguinis*, only for 10%NPs group. Similar results were showed in other NPs such as ZnO, chitosan and the combination of ZnO and chitosan, compared to TiO_2_. They showed that NPs maintain their properties for a long time, up to three weeks, since just a slight increase in the number of colonies was observed, unlike *L. acidophilus* that by increasing time the number of colonies remains constant.^19^ ^,^ ^22^ ^,^ ^23^

Composites containing TiO_2_ nanoparticles have a significantly different shear bond strength from that of the original Transbond XT composites - used as gold standard. The bond strength decreased as the percentage of nanoparticles increased in the composite. The highest mean SBS belonged to the control group, while the lowest value was seen in 10% NP composite group. However, there was no significant difference between the obtained shear bond strength values in composite containing less than 5% nanoparticles and they were within the acceptable range of 6-8Mpa. However this may not be true for 10% NP group.

These results are in contrast to the results of other^26^ study that showed equal bond strengths in the composites containing TiO_2_ (standard P_25_) and the control group. They just evaluated 1% nanoparticles incorporated into the composite. They concluded that adding TiO_2_ nanoparticles to orthodontic composite couldn’t compromise the shear bond strength and debonding pattern of composite resins.^26^ Similar results were obtained by Elsaka et al.^30^ after incorporating TiO_2_ nanoparticles to glass-ionomer powder. They showed 3% concentration of TiO_2_ improved mechanical properties of composite resins.

## CONCLUSION

Incorporating TiO_2_ nanoparticles into composite resins confer antibacterial properties to adhesives, while the mean shear bond of composite containing 1% and 5% nanoparticles still in an acceptable range.
